# The power of rotation schedules on the career selection decisions of medical students

**DOI:** 10.1007/s10459-023-10227-w

**Published:** 2023-05-02

**Authors:** John P. Bechara, Priti Pradhan Shah, Keith Lindor

**Affiliations:** 1 Department of Organization Studies, Tilburg University, Simon Building, PO Box 90153, Tilburg, 5000 LE The Netherlands; 2 Work and Organizations, Carlson School of Management, University of Minnesota, Twin Cities, Minneapolis, MN USA; 3 Mayo Clinic College of Medicine, Mayo Clinic, Rochester, MN USA

**Keywords:** Career selection decisions, Medical students, Clinical rotations, Timing, Duration

## Abstract

Choosing a career pathway in medicine is a high stakes decision for both medical students and the field of medicine as a whole. While past research has examined how characteristics of the medical student or specialties influence this decision, we introduce temporal elements as novel variables influencing career selection decisions in medicine. Specifically, we investigate how timing and duration of residency options, based on a rotation schedule that medical students have limited control over, influence their career selection decisions. An archival study investigating 5 years of medical student rotation schedules (N = 115) reveals that clinical rotation options appearing earlier and more often in the schedule were more likely to be selected. Moreover, timing and duration of exposure interacted such that residency options appearing later in the schedules were more likely to be selected if they also appeared more often. Conditional logistic regressions using student fixed-effects to control for idiosyncratic medical student differences (i.e., gender, & debt, etc.), and residency fixed-effects to control for idiosyncratic residency differences (income, and lifestyle, etc.), revealed the rotation schedule had a significant impact on residency selection decisions even when controlling for factors typically influencing this decision. Medical students’ career decisions are influenced by when and how long different choice options appear in their rotation schedule, especially when they have limited influence over this schedule. The results have implications for healthcare policy by highlighting a tool for adjusting physician workforce composition by broadening exposure to a greater array of career options.

## Introduction

Choosing a career pathway in medicine is a high stakes decision for both medical students and the field of medicine as a whole. For medical students, selecting a residency has long term consequences impacting their future opportunities, income and lifestyle. In medicine, the career choices made by medical students impact the composition of the physician workforce and access to care for the general population (Clinite et al., 2014; West & Dupras, 2012). As such, scholars have investigated numerous factors influencing this important decision, ranging from *characteristics of medical students* such as: admission scores (Ward et al., 2004), gender (Newton et al., 1998; van der Horst et al., 2010; Ward et al., 2004), marital status (Newton et al., 1998), debt (Bland et al., 1995; Grayson et al., 2012), interests and values (Newton et al., 1998), parents’ careers (Griffin & Hu, 2019; Ward et al., 2004), personality traits (Lepièce et al., 2016; Ward et al., 2004); *characteristics of specialties* such as: training requirements (Dorsey et al., 2003; van der Horst et al., 2010), income (Dorsey et al., 2003; Grayson et al., 2012), work hours (Dorsey et al., 2003), lifestyle (Clinite et al., 2014; Dorsey et al., 2003; Lefevre et al., 2010; Schwartz et al., 1995), patient problems (Lefevre et al., 2010; van der Horst et al., 2010), prestige (Newton et al., 1998); and *characteristics of students’ experience in a specialty* such as: exposure (Bland et al., 1995; Dobie et al., 1997; Maiorova et al., 2008; Mihalynuk et al., 2006), satisfaction (Schwartz et al., 1995), quality of patient relations (Ellsbury et al., 1998), and quality of teachers (Griffith et al., 2000; van der Horst et al., 2010).

Absent from this body of work is the role of temporal elements over which students may have limited control, such as timing and duration of access to career related information. Specifically, medical students’ clinical rotation schedules, exogenously determines the timing in which residency information is accessed and the duration of exposure to such information (Shah et al., 2013). However, the influence of these temporal factors on medical students’ career selection decisions has yet to be considered.

Given the importance of residency selection decisions, this body of work typically focuses on a rational purposeful decision-making process where students weigh the various factors to obtain the best match between their attributes and that of the specialty (Bland et al., 1995; Pfarrwaller et al., 2017) also known as subjective expected utility theory (Gorenflo et al., 1994; Reed et al., 2001). According to subjective expective utility theory, three core principles drive decisions: subjective value of options available, probability of obtaining the option, and the interaction of these two factors (Hastie & Dawes, 2009; Lepièce et al., 2016; Reed et al., 2001; Von Neumann & Morgenstern, 1947). More specifically, it is assumed that medical students know all their available options and have well-defined preferences among these options. Students are also aware of their probability of obtaining each option based on their own credentials and the availability or competitiveness of residency positions. Last, students weigh the combination of their own preferences across different residency options with the likelihood of obtaining these options to determine the optimal fit. For example, a competitive residency that is highly valued by a medical student may be preferred to one that is easier to obtain, but less valued. Importantly, these internal calculations to determine subjective expective utility are individualized resulting in different preferences across residencies for each student.

Rational decision-making frameworks are popular when investigating medical students’ career decisions as all the elements needed to promote rational decision making are available. The decision is high-stakes and not easily reversed (i.e., this decision determines future opportunities, lifestyle or income and switching costs are high). All options are known (i.e., 26 residencies). Options are distinct (i.e., residencies focus on different organs, diseases, or medical problems). Information about options and the probability of obtaining options is accessible and abundant (i.e., via medical school training, advising, and the American Medical Association website) (Bazerman & Moore, 2012; Shah et al., 2013). Under these conditions, the aforementioned temporal elements would be unlikely to influence the career selection of medical students. However, we draw from the decision bias literature to illustrate that both these temporal factors can influence even decisions of this magnitude.

We propose an alternative approach to residency selection decisions where decision-makers are assumed to have limited capacity for processing information and computation. Rooted in the decision bias literature (Bettman et al., 1998) which construes students as constructing their preferences for different options based on their interaction and experience with different options. As such, these temporal elements are likely to play a crucial role in shaping students’ career selection decisions. In the next sections, we develop these ideas further.

### The role of timing of first exposure on career selection decisions

While not typically investigated in medical career decision making, timing is an important factor in socialization, preference formation and decision making research. Socialization research finds that there is an early window of time when individuals are more susceptible to influence. Individuals transitioning into a new career are adjusting to new role demands resulting in heightened uncertainty, greater awareness of their external environment and reduced sense of self (Ibarra & Andrews, 1993; Pratt et al., 2006). Taken together, this experience results in greater receptivity and susceptibility to external influence. In the decision making domain, primacy research finds that we focus upon early information and discount that which comes later, resulting in a disproportionate influence of the former on our preferences and decisions, even in the context of medical decisions (Curley et al., 1988). Based on the belief-updating model, early information anchors our judgements and helps shape our preferences, while later information fails to get noticed or encoded as it lacks novelty or is assimilated based on preceding information (Hogarth & Einhorn, 1992; Tversky & Kahneman, 1974). Given these dual drivers, recent research on career selection decision of residents finds that subspecialty decisions are significantly influenced by rotations first experienced early in a resident’s schedule (Shah et al., 2013). In effect, rotations first experienced early in a medical student’s schedule are apt to be particularly influential especially as students are more susceptible to external information in the early stages of their career (Smith et al., 1997). More formally, we hypothesize that the timing of first exposure to a residency option during medical students’ clinical rotations will influence their career selection decisions such that medical students are more likely to select residency options they experience earlier in their clinical rotations, even when controlling for relevant attributes of the options.

### The role of duration of exposure on career selection decisions

In addition to the timing of first exposure, we also investigate how the duration of exposure to residency options influences residency selection decisions. Duration of exposure is defined as the amount of time medical students experience a rotation. Past research on medical career decisions does suggest that exposure to a profession plays a role in generating interest in and even selecting an area (Berman et al., 2012; Maiorova et al., 2008; Parlier et al., 2018; Shah et al., 2013; Yamane et al., 2020). Much of the earlier work provides a systematic review of the literature, case analysis or focuses on exposure to one or just a handful of specific residency options (Bland et al., 1995; Dobie et al., 1997; Maiorova et al., 2008; Mihalynuk et al., 2006); however, our study is the first to investigate the influence of exposure across all residency options available to medical students, while simultaneously controlling for both idiosyncratic characteristics of the residency options and students. We argue that greater exposure with a residency option provides an opportunity to reduce uncertainty as more knowledge is gained, more skills are learned and there is greater opportunity to assess fit with an option.

Decision making research finds that when access to information is not consistent across options, people often favor options in which they have greater experience or knowledge (Le Mens & Denrell, 2011). Career selection decisions in medicine are often plagued with uncertainty as residents feel uncomfortable settling on a choice and 62% of residents change their career plans at least once prior to the application deadline (Smith et al., 1997; West & Dupras, 2012). Uncertainty in career selection decisions can be based on inadequate understanding, incomplete information, conflicting information and undifferentiated alternatives (Lipshitz & Strauss, 1997). Greater exposure to a residency option results in greater awareness of and certainty about the merits and demerits associated with a residency option and the consequences of selecting the residency (Shah et al., 2013). In sum, we hypothesize that the duration of a residency option during medical students’ clinical rotations will influence their career selection decisions such that medical students are more likely to select residency options they experience more often in their clinical rotations, even when controlling for relevant attributes of the options.

### The interactive effect of timing of first exposure and duration of exposure on career selection decisions

While it is likely that medical students will select residency options that first appear early and more often over those that first appear later and less often, what happens when a residency option first appears later but for a greater period of time or early but for a shorter period of time? The initial anchor may be established when a residency option appears early in a medical student’s rotation schedule; however, if the experience is limited, the anchor may be less durable and susceptible to adjustment given the limited information (Shah et al., 2013). In contrast, greater duration of experience with a residency option appearing later provides more abundant information that is apt to mitigate the initial anchor of earlier appearing residency options. As such we hypothesize that the duration of and timing of first exposure to a residency option during medical students’ clinical rotations will interact to influence their career selection decisions such that medical students are more likely to select residency options they experience later and more often in their clinical rotations, even when controlling for relevant attributes of the options.

## Methods

### Sample

The sample included all 134 medical students who graduated between 2002 and 2004 at the Mayo Medical School (MMS) in Rochester, Minnesota. Of these medical students 115 (86%) were included in our final sample. Students were excluded if their third or fourth year rotation schedules were not available, they took a transitional year, repeated a year, or were completing dual degree (MD-PhD). Students could select from one of 26 residency options upon graduation. The sample consists of 2990 medical student-residency observations (115 students x 26 residency options).

### Ethics

This study was approved by the University of Minnesota Institutional Review Board.

### Setting

The MMS is a research-oriented medical school. Consistently ranking among the best medical schools in the United States (*How Does Mayo Clinic School of Medicine (Alix) Rank Among America’s Best Medical Schools?*, n.d.), it is also one of the most selective medical schools (*10 Med Schools With the Lowest Acceptance Rates*, n.d.). Similar to other U.S. medical schools, most MMS students spend their third- and fourth-year in a hospital setting experiencing the different residency options as part of the medical curriculum. More importantly for our research, medical students graduating between 2002 and 2004 had no input in their third-year clinical rotation schedule and had very limited choice in their fourth-year clinical schedule. Medical students had very few (1 to 2) elective options in their fourth year, the timing of which was typically determined by their research rotations based on their advisors’ schedules. Thus, students can select their research area, but not when they can conduct the research in their schedule. The remaining rotations and one or two electives are placed around this research rotation.

### Data sources

We used 5 years of third- and fourth-year rotation schedules of medical students at MMS. Third- and fourth-year clinical rotations were the first opportunity for students to experience the residency options in a hospital setting. Data on the actual residency options selected by students were obtained from MMS.

### Covariates

As all options that medical students can select from are known and students can only select one of the 26 available residency options, we can control for factors typically unaccounted for in career selection decisions. Covariates included residency options fixed-effects which control for unobserved time invariant differences across residency options. These include, but are not limited to, salary, controllability of lifestyle, prestige, popularity, abundance of positions, base rate for training exposure, competitiveness of acquiring a residency and overall attractiveness of future job alternatives. We also included student fixed-effects which control for unobserved time invariant student differences, including, but are not limited to, gender, values, parents’ careers, personality traits among other differences. See Table 1 for more information about the covariates.

**Table 1:** Variable Descriptions

Variable	Description	Type
Timing of first exposure	Coded as the week a student was first exposed to a residency (1 = first week, 2 = second week…, 100 = last week, and 101 = no exposure)	Continuous
Duration of first exposure	Coded as the total number of weeks a student was exposed to a residency (0 = no exposure, 1 = one week, 2 = 2 weeks…)	Continuous
Career selection	Coded as 1 if a student selected a specialty from the 26 available residency options and 0 = otherwise	Binary
Residency fixed-effects	Coded 25 binary variables indicating 1 = for each residency and 0 = otherwise except for Anesthesiology which was the base specialty. Controls for residency attributes such as salary, controllability of lifestyle, prestige, popularity, abundance of positions, base rate for training exposure, competitiveness of acquiring a residency and overall attractiveness of future job alternatives	Binary
Student fixed-effects	Coded 114 binary variables indicating 1 = for each individual and 0 = otherwise. Controls for medical student attributes such as gender, values, parents’ careers, personality traits among other differences.	Binary

### Independent and outcome variables

The independent variables are timing of first exposure to a residency and duration of exposure to a residency. Timing of first exposure was coded as the first week a student was exposed to a residency. For example, timing for pediatrics for a student first exposed to it in the 8th week of their rotation schedule was coded as 8. Duration of exposure was coded as the total number of weeks exposed to a residency. For example, if a student was exposed to surgery for 5 weeks during their rotation schedule, the exposure for surgery was coded as 5. See Table 1 for more information about the independent variables.

The outcome variable, career selection, is a binary variable indicating 1 = for the selected option and 0 = for the 25 non-selected options.

### Analytical plan

Descriptive statistics, including means and standard deviation, were calculated for each variable. We compared the timing of first exposure and the duration of exposure for both selected and non-selected residency options overall and across all residency options and for each of the different residency options using two-group *t* tests with an alpha = 0.05. We used the conditional logistic regression to test the effect of timing of first exposure and the duration of exposure on career selection decisions. The conditional logistic regression is suitable for data on selection decisions when we have variables on the characteristics of the entire set of career choices. This method estimates the effect of a change in a residency-student attribute on the likelihood of selecting a particular residency. The data were analyzed using Stata SE 17.0 for Windows (Stata Corp, College Station, Texas).

## Results

### Descriptive statistics

Table 2 shows (unadjusted) differences between selected and non-selected residency options across all residency options. Results reveal significant differences in timing and duration between selected and non-selected residency options. Medical students selected residency options they were exposed to earlier in their rotation schedule (39th week vs. 77th week) and for much longer periods of time (10 weeks vs. 2 weeks) compared to those that were not selected. Table 3 provides information about the distribution of selected residency options. In effect, when medical students select a residency option, they are making 26 separate decisions. They are selecting one option and rejecting the 25 other options. Table 3 shows the three most frequently selected residency options were pediatrics (17 students), internal medicine (13 students), and anesthesiology (13 students). Additionally, Table 3 shows (unadjusted) differences between selected and non-selected residency options for each of the 17 different residency options that were selected by at least one medical student and were experienced during their third or fourth year. Results reveal significant differences in timing of first exposure for 10 of 17 residency options selected and experienced. For example, medical students selecting Anesthesiology, Dermatology, Emergency Medicine, Ophthalmology, Orthopedic Surgery, Otolaryngology, Pathology, Pediatrics, Radiation Oncology and Urology, were significantly more likely to be exposed to these rotations early in their clinical schedule compared to the residency options they did not select. Results provide robust support for duration of exposure with medical students having significantly greater exposure to all selected residency options compared to those that were not selected.

**Table 2:** Descriptive Statistics for Timing of First Exposure and Duration of Exposure for Selected and Non-Selected Residency options for Medical Students at the Mayo Medical School ^a^

	Selected residency		Non-selected residency		Selected vs. non-Selected
	Mean (SD)		Mean (SD)		P value
Timing of first exposure	38.94 (23.75)		76.62 (34.15)		0.00
Duration of exposure	10.02 (5.79)		2.02 (3.25)		0.00

^a^ Comparison of timing and duration for selected and non-selected residency options employed 2-group t-tests with df = 2990. Bold indicates significance, P < .05

**Table 3:** Descriptive Statistics for Timing of First Exposure and Duration of Exposure Across all 17 Residency Options Selected by Medical Students at the Mayo Medical School ^a^

	Selected residency options			Non-selected residency options		Selected vs. non-Selected Timing	Selected vs. non-Selected Duration	
Residency name ^b^	N (%)	Timing Mean (SD)	Duration Mean (SD)	Timing Mean (SD)	Duration Mean (SD)	P value	P value	
Anesthesiology	13 (11%)	**51.41(13.47)**	**6.88 (2.42)**	**93.86 (13.89)**	**0.66 (1.25)**	**0.00**	**0.00**	
Dermatology	6 (5%)	**64.66 (17.88)**	**4.16 (2.63)**	**97.51 (10.10)**	**0.40 (1.08)**	**0.00**	**0.00**	
Emergency Medicine	7 (6%)	**32.00 (27.19)**	**8.14 (3.76)**	**76.97 (15.94)**	**3.46 (2.03)**	**0.00**	**0.00**	
Family Medicine	8 (7%)	33.12 (19.037)	**7 (2.82)**	32.76 (12.64)	**2.37 (1.17)**	0.94	**0.00**	
General Surgery	7 (6%)	17.42 (12.90)	**10 (3.26)**	24.98 (15.41)	**6.83 (1.64)**	0.20	**0.00**	
Internal Medicine	13 (11%)	28.53 (15.31)	**18 (3.97)**	27.08 (14.16)	**12.36 (3.49)**	0.73	**0.00**	
Neurology	3 (3%)	38.33 (22.30)	**5.66 (2.30)**	24.64 (15.85)	**3.17 (0.76)**	0.14	**0.00**	
Obstetrics and Gynecology	8 (7%)	30.87 (15.92)	**11.88 (3.51)**	25.14 (15.79)	**6.39 (1.14)**	0.32	**0.00**	
Ophthalmology	3 (3%)	**55.50 (45.50)**	**6.33 (5.68)**	**97.56 (8.59)**	**0.35 (0.91)**	**0.00**	**0.00**	
Orthopedic Surgery	2 (2%)	**62.00 (7.07)**	**12 (0.00)**	**97.45 (9.43)**	**0.47 (1.13)**	**0.00**	**0.00**	
Otolaryngology	2 (2%)	**58 (1.41)**	**8.00 (1.41)**	**97.58 (9.54)**	**0.44 (1.10)**	**0.00**	**0.00**	
Pathology	3 (2%)	**59.00 (6.08)**	**4.00 (1.00)**	**97.84 (10.12)**	**0.31 (0.97)**	**0.00**	**0.00**	
Pediatrics	17 (15%)	**21.64 (12.80)**	**16.47 (5.18)**	**24.93 (15.77)**	**7.35 (2.84)**	**0.00**	**0.00**	
Psychiatry	3 (3%)	32.66 (11.37)	**11.80 (4.20)**	28.25 (13.86)	**4.26 (0.97)**	0.58	**0.00**	
Radiation Oncology	3 (3%)	**61.00 (3.46)**	**8.33 (1.52)**	**99.81 (6.75)**	**0.09 (0.53)**	**0.00**	**0.00**	
Radiology	12 (10%)	35.83 (28.16)	**7.41 (2.96)**	40.09 (32.99)	**3.30 (1.72)**	0.66	**0.00**	
Urology	3 (3%)	**47 (13.85)**	**8.66 (5.03)**	**99.27 (7.47)**	**0.25 (1.00)**	**0.00**	**0.00**	

^a^ Comparison of timing and duration for selected and non-selected residency options employed 2-group t-tests with df = 113. Bold indicates significance, P < .05. We only show the residency options where at least one medical student selected it.

^b^ Two students selected Internal Medicine/Pediatrics however, we did not include the residency in the table since they were never exposed to this residency in their rotation schedule.

### Regression model results

Results of the conditional logistic regression predicting career selection decisions, adjusted for, residency fixed-effects and student-fixed effects, are provided in Table 4. The results reveal a significant negative effect of timing of exposure *(β*_*Timing*_*= -0.02, p < .05)* providing support for our prediction that earlier first exposure to a residency option increases the likelihood of selecting that option. Additionally, the results show a significant positive effect of duration of exposure on selection decisions *(β*_*Duration*_ *= 0.52, p < .01)* providing supporting for our prediction that longer exposure to a residency option increases the likelihood of selecting that option. Finally, the results also reveal a significant interaction between timing of first exposure and duration of exposure *(β*_*Timing X Duration*_ *= 0.003, p < .05)* providing support for our last prediction about longer and later exposure to a residency option increases the likelihood of selecting that option. Notable, our results for timing and duration of exposure and the interaction of these two variables are significant even when controlling for characteristics of the medical students and the residency options examined in past research using our fixed-effects specification.

**Table 4:** Results of Conditional logit Regression Model for Career Residency Selection Decisions among Medical Students at the Mayo Medical School ^a^

				95% CI for OR		
Coefficient	β	(SE)	*P*	OR	LB	UB
Timing of first exposure	-0.02	(0.01)	0.02	0.98	0.96	1.00
Duration of exposure	0.52	(0.071)	0.00	1.69	1.46	1.95
Timing of first exposure X Duration of exposure	0.003	(0.001)	0.01	1.003	1.001	1.01

Abbreviations: SE indicates standard error; OR, odds ratio; CI, confidence interval; LB, lower bound; UB, upper bound; UP.

^a^ N = 2990 medical student-residency observations based on 115 students. Selection decisions coded 1 = yes, 0 = no. Conditional logistic regression with standard predictor entry used. Overall model fit χ2(3) = 182.54, P < .0001, Log-likelihood = -153.44. Covariates included but not shown: 25 residency fixed-effects and 114 medical student fixed-effects. Bold indicates significance, P < .05.

**Fig. 1:**
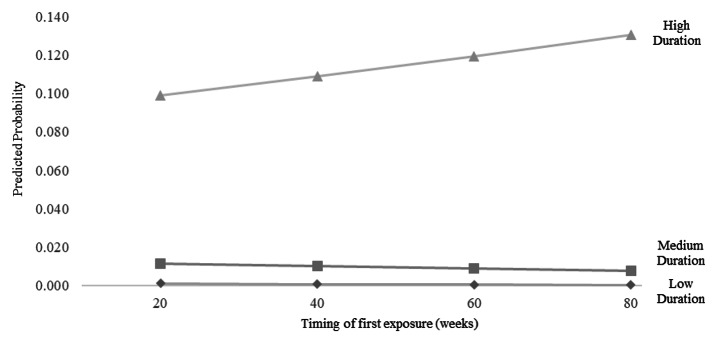
Predicted Probability of Selecting Pediatrics at Different Levels of Timing of First Exposure and Duration of First Exposure ^a^

^a^ We chose pediatrics since it was the most frequently selected residency in our sample. Low duration refers to 2 weeks of exposure, medium duration refers to 6 weeks of exposure, and high exposure refers to 10 weeks of exposure.

To provide additional insight into the nature of this interaction, we plotted the effect of timing of first exposure by duration of exposure in Fig. 1. The x-axis refers to the timing of first exposure (in weeks), the y-axis refers to the predicted probability of selecting a residency (Pediatrics in this case) and each line conveys the effect at different levels of duration of exposure (in weeks). The figure reveals that earlier first exposure had a very small increase on the likelihood of selection at low and medium levels of duration.

of exposure. However, when the option was experienced for a greater amount of time, later exposure had a large increase on the likelihood of selection.

## Discussion

The results provide ample evidence that temporal elements, currently ignored in the research, play an important role in career selection decisions in medicine. Specifically, the results illustrate how a rotation schedule which medical students have minimal control over can influence their residency selection decisions. The timing and duration of exposure to residency options in a rotation schedule, influences medical students’ career selection decisions, even when adjusting for unobserved attributes of the residency option and the medical student providing support for our hypothesized claims. Medical students have lower odds (0.98) of selecting a residency they were first exposed to one week later. Put differently, medical students select residency options that appear earlier in their rotation schedule. Medical students have a higher odds (1.69) of selecting a residency option they were exposed to for one more week. In other words, medical students select residency options they were exposed to for a longer duration of time in their rotation schedule. Finally, medical students have a higher odds (1.003) of selecting a residency option they were first exposed to one week later and one week longer. Otherwise stated, medical students select residency options appearing later in their rotation schedule if they also appear more often. The findings provide evidence for the importance of a variable influencing medical students’ career selection decisions that has to date been ignored, yet has tremendous potential as it is in the control of the medical schools.

The mean differences between selected and non-selected options provide a window into the optimal time period when medical students are most likely to be influenced by their schedule. Specifically, on average, medical students were more likely to select residency options that they experienced in their first 39 weeks of their rotation schedule and those that they experienced for on average 10 weeks. While earlier research has suggested that prior exposure is an important determinant influencing the decision to enter primary care, this work was based on a systematic review of the literature or focused on just exposure to primary care or a handful of residency options while not accounting for exposure to other residency options (Berman et al., 2012; Maiorova et al., 2008; Newton et al., 1998; Parlier et al., 2018; Shah et al., 2013). In contrast, our analysis accounts for exposure to all other residency options, while simultaneously controlling for both idiosyncratic characteristics of the residency options and students. Moreover, the findings highlight the timeframe needed for exposure to influence residency selection decisions.

Importantly, our results are adjusted for covariates examined by prior work using our fixed-effects specification. Our residency fixed-effects account for residency specific attributes, such as salary, controllability of lifestyle, prestige, popularity, abundance of positions, base rate for training exposure, competitiveness of acquiring a residency and overall attractiveness of future job alternatives. Our student fixed-effects account for medical student specific attributes, such as gender, values, parents’ careers, personality traits among other differences. In doing so, we can be certain that the results presented are due to the timing and duration of exposure to residency options, even when controlling for the other aforementioned factors.

Our findings have important implications for medical education. The results illustrate the hazards associated with limited scheduling input from medical students. When rotation order and duration of exposure to residency options are predetermined, these factors drive career selection decisions and may have unintended negative consequences. Specifically, medical students may be less likely to explore career pathways in residency options appearing later or less frequently, precluding them from possibly finding residency options that better fit their skills, expertise, or preferences. Moreover, an important component of medical education is to provide students with exposure to a wide array of experiential training opportunities representative of the field of medicine. Our findings suggest that as the window for exploration closes in the early stage of decision-making, medical students’ preferred choices may ossify, inhibiting them from fully experiencing and appreciating the benefits associated with training opportunities appearing later. Overall, the findings provide a rationale for increasing exposure to a broader range of options, providing elective options, and providing some control over when these residency options are experienced. Otherwise, career choice may be influenced by an arbitrary sequence or duration of rotations.

Our findings also have important implications for healthcare policy. The results highlight an additional tool for adjusting the composition of the physician workforce. Due to our aging population, the demand for primary care physicians continues to outpace the supply. The Association of American Medical Colleges (AAMC) estimates a shortfall of between 21,400 and 55,200 primary care physicians by 2033 (*The Complexities of Physician Supply and Demand: Projections From 2018 to 2033*, 2018). Petterson and colleagues similarly estimate more than a 44,000 deficit extrapolating to 2035 (Petterson et al., 2015). As witnessed in our current response to the Covid-19 pandemic, a robust primary care infrastructure is integral for a high functioning health system. Existing research focuses on monetary incentives to rectify this imbalance using scholarships, tuition reimbursements, loan remission and signing bonuses (De et al., 2004; Pathman et al., 2000). In contrast, our findings suggest altering the timing and duration of exposure to primary care residency options or other underrepresented residency options provide an alternative non-monetary way to alter the composition of the physician workforce. Notably, the rotation schedule upon which these variables are based are already in the control of medical institutions.

A strength of this study is our use of archival data on medical students’ rotation schedules to construct our core temporal variables of timing of first exposure and duration of exposure. Additionally, given the fixed universe of career options, the inclusion of selected and non-selected residency options, and adjusting for residency and student idiosyncratic differences with our fixed-effects, we provide rigorous yet conservative empirical test of our claims. Even so, there are limitations associated with this research. The archival data does not enable us to determine quality of experience or the mechanism of influence based on the timing of first exposure and duration of exposure. The data was also collected in one medical center, which could limit generalizability, albeit, the lack of choice in a medical rotation schedule is common across other institutions.

In summary, our findings add to the existing body of research on career selection decisions in medicine by highlight an important yet omitted temporal variable influencing medical students’ career selection decisions. The timing and duration of exposure to residency options impact career selection decisions. Importantly, while medical students have limited control over the schedule that determines these temporal elements, it is under the control of the medical institutions in which they are trained.
